# A Genome-Wide Scan Reveals Important Roles of DNA Methylation in Human Longevity by Regulating Age-Related Disease Genes

**DOI:** 10.1371/journal.pone.0120388

**Published:** 2015-03-20

**Authors:** Fu-Hui Xiao, Yong-Han He, Qi-Gang Li, Huan Wu, Long-Hai Luo, Qing-Peng Kong

**Affiliations:** 1 State Key Laboratory of Genetic Resources and Evolution, Kunming Institute of Zoology, Chinese Academy of Sciences, Kunming, Yunnan Province, China; 2 KIZ/CUHK Joint Laboratory of Bioresources and Molecular Research in Common Diseases, Kunming, Yunnan Province, China; 3 University of Chinese Academy of Sciences, Beijing, China; 4 Beijing Genome Institute at Shenzhen, Shenzhen, China; Harbin Medical University, CHINA

## Abstract

It is recognized that genetic factors contribute to human longevity. Besides the hypothesis of existence of longevity genes, another suggests that a lower frequency of risk alleles decreases the incidence of age-related diseases in the long-lived people. However, the latter finds no support from recent genetic studies. Considering the crucial role of epigenetic modification in gene regulation, we then hypothesize that suppressing disease-related genes in longevity individuals is likely achieved by epigenetic modification, e.g. DNA methylation. To test this hypothesis, we investigated the genome-wide methylation profile in 4 Chinese female centenarians and 4 middle-aged controls using methyl-DNA immunoprecipitation sequencing. 626 differentially methylated regions (DMRs) were observed between both groups. Interestingly, genes with these DMRs were enriched in age-related diseases, including type-2 diabetes, cardiovascular disease, stroke and Alzheimer’s disease. This pattern remains rather stable after including methylomes of two white individuals. Further analyses suggest that the observed DMRs likely have functional roles in regulating disease-associated gene expressions, with some genes [e.g. caspase 3 (*CASP3*)] being down-regulated whereas the others [i.e. interleukin 1 receptor, type 2 (*IL1R2*)] up-regulated. Therefore, our study suggests that suppressing the disease-related genes via epigenetic modification is an important contributor to human longevity.

## Introduction

Human longevity is believed to be an integrating result of genetic and environmental factors. Although previous studies have shown that genetic variation may explain 20–30% contribution to human longevity [1, 2], much remains to be known for its underlying genetic mechanism. In the past decade, a number of genes, e.g. Protein DAF-2 (*daf-2*), Protein DAF-16 (*daf-16*), Protein SIR-2 (*sir-2*) [3–5], were discovered, in which some specifically genetic alterations may confer advantage in extending the organisms’ lifespan, suggesting the existence of longevity genes. These findings however could not fully explain the significantly reduced incidence of age-related diseases in centenarians and their offspring [6–8], as it requires a broad effect of longevity genes, including conferring beneficial effects in extending life span as well as suppressing deleterious influence from the disease-associated genes. Alternatively, it is possible that the low prevalence of the age-related diseases in the long-lived people is attributed to a much lower frequency of risk alleles [9]. Unfortunately, the latter notion fails to find support from a recent study in which the long-lived people were shown to carry similar frequencies of risk alleles as did in the young controls [10]. This observation seems to echo with the suggestion that the longevity-related variants may compress the morbidity of long-lived people as these variants were significantly enriched in disease-related genes [11].

Hitherto, the obtained genetic evidence, based virtually on mutation screening, find no support for the hypothesis that lack of disease-related mutations contributes to healthy aging. However, taking into account the heterogeneity in longevity, in which multiple ways could be adopted to achieve longevity [12], and the crucial role of epigenetic modification in gene regulation, we hypothesize that suppressing the disease-related genes in the longevity individuals is likely achieved by the epigenetic modification, e.g. DNA methylation. Indeed, DNA methylation, mainly by way of a methyl group to the 5-position of cytosine (5m-C), plays an important role in regulating gene expression [13]. Meanwhile, a reduction of genome-wide DNA methylation level and locus-specific hyper-methylation did have been observed with aging [14]; whereas changes in DNA methylation were reported to be associated with the occurrences of age-related diseases, such as cardiovascular disease, diabetes and cancer [15–17].

To test our hypothesis, the genome-wide landscapes of DNA methylation were obtained by using methyl-DNA immunoprecipitation sequencing (MeDIP-Seq) [18]. Comparison between the Chinese female centenarians and middle-aged controls led to the identification of a number of the differentially methylated regions (DMRs). Our further analyses revealed that the identified DMRs were significantly enriched in genes associated with age-related diseases, and this pattern remained rather stable after the epigenetic genomes from two white individuals [19] were included, arguing for the notion that DNA methylation may contribute to healthy aging in human populations by regulating the genes that confer susceptibility to the age-related diseases.

## Results

### DNA methylation landscapes across centenarians and middle-aged individuals

**Fig 1:**
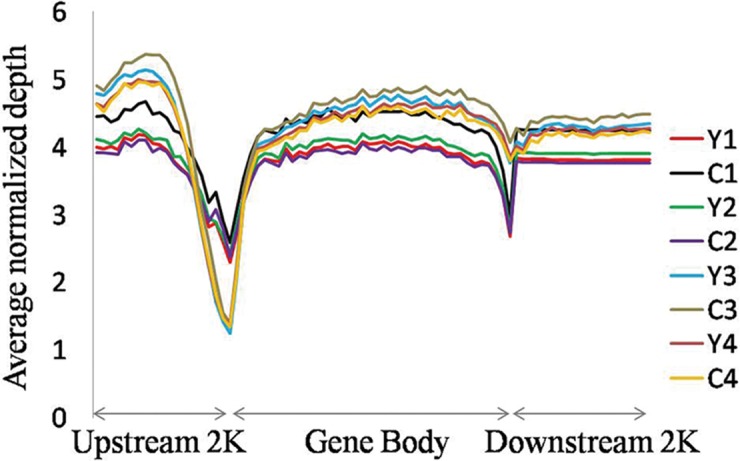
Relative methylation levels of different human genomic sequences in the centenarians and controls. The relative methylation level of each genomic sequence was calculated based on the reads numbers that aligned on loci in human genome. The gene-body sequences were split into 40 non-overlap windows, up- and down-stream genomic sequences were split into 20 non-overlap windows and the mean relative methylation level was calculated for each window. (Y and C represent the younger control and centenarian samples, respectively).

In this study, DNA methylation profiles of 4 female healthy centenarians and 4 ethnicity matched middle-aged individuals were obtained by using the MeDIP-Seq method. More than 60 million uniquely mapped paired-end reads were produced. Saturation and coverage analysis indicated that the produced data have sufficient reads to generate a reproducible genome-wide methylation profile for each sample (S1 Fig.) and cover more than 80% CpGs in human genome (S2 Fig.) [20]. As shown in Fig. 1, DNA methylation signal decreased sharply before the transcription start site and increased considerably towards the gene body regions and then was maintained at a plateau until the end of the gene body. This uneven pattern suggests potential roles of methylation in the regulation of gene expression depending on their location.

### Numerous differentially methylated regions (DMRs) exist between the centenarians and controls

**Fig 2:**
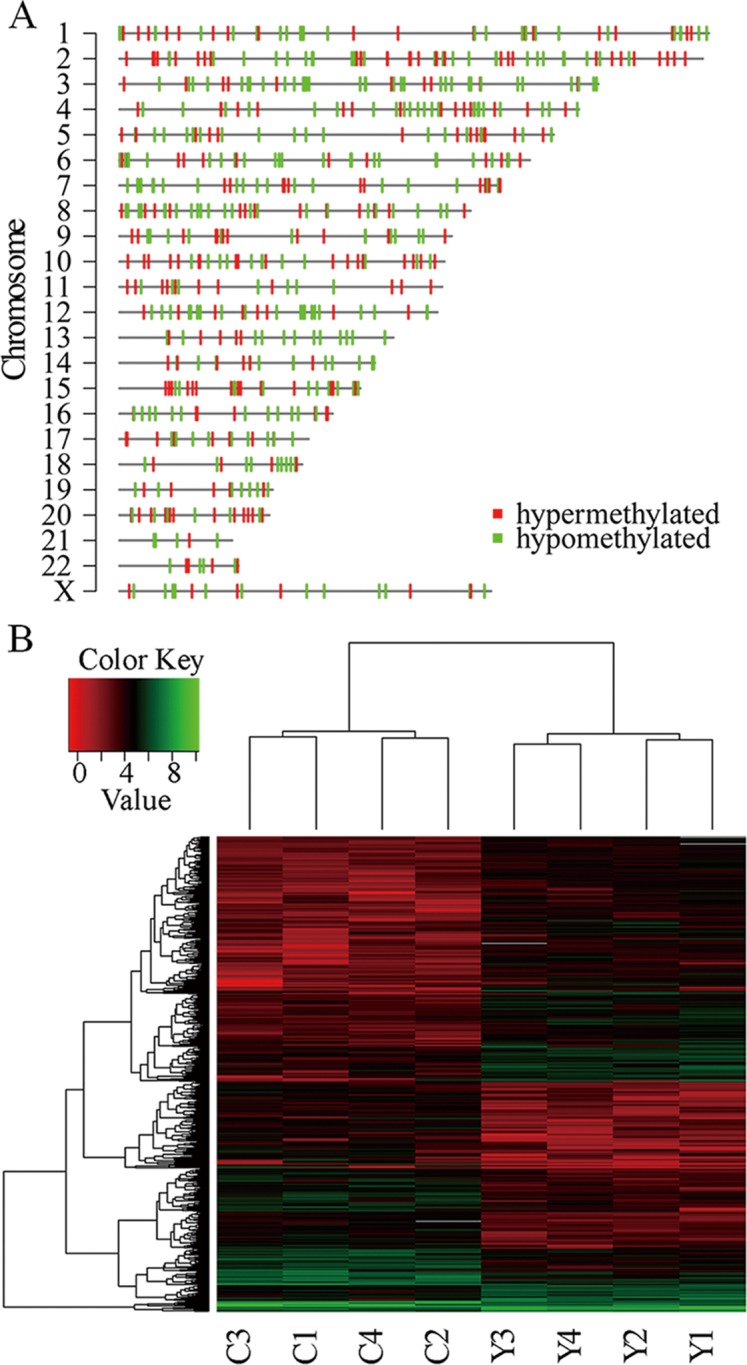
The identified differentially methylated regions (DMRs). (A) Vision of the distribution of DMRs on each chromosome. (B) Heatmap of all DMRs (The methylation value of each DMR was log2 transformed).

**Fig 3:**
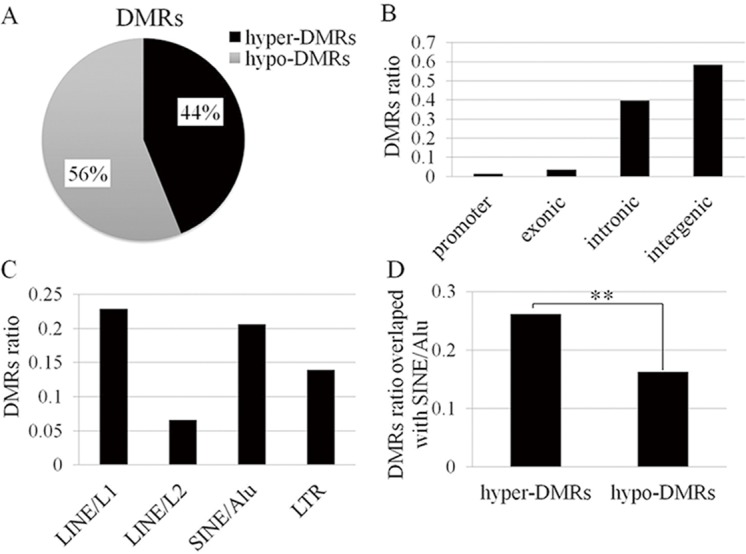
DMRs ratio. (A) The percentage of hyper-DMRs and hypo-DMRs. (B) DMRs ratio overlapped with different genomic sequences. (C) DMRs ratio overlapped with different repeat elements. (D) The hyper-DMRs are preferentially overlapped with SINE/Alu repeat compared with hypo-DMRs (** p < 0.01).

A total of 887 segments showed significantly different methylation status between the centenarians and controls (*p* < 0.0005). Then 626 differentially methylated regions (DMRs) were identified by leaving those segments with CpGs and merging the adjacent segments with the same direction of DNA methylation change, which seem to have random distribution on each chromosome (Fig. 2A). The FDR of each identified DMR was estimated using permutation test in which the maximum FDR was 6.3% (1000 permutations). The heatmap showed that the identified DMRs were able to separate the samples into younger and longevity group (Fig. 2B). Among these DMRs, 274 (44%) and 350 (56%) were hypermethylated and hypomethylated respectively in centenarians in comparison with the younger controls (Fig. 3A, S1 Dataset).

The DMRs were found to locate in promoter (1.3%), exonic (3.5%), intronic (39.8%) and intergenic (58.5%) regions (Fig. 3B). The most common repetitive sequences were LINE 1 repeats (22.8%), Alu repeats (20.6%), LINE 2 repeats (6.5%), and LTR-retrotransposons (13.9%) (Fig. 3C). It should be noted that the preferential hypermethylated DMRs were overlapped with Alu sequences compared with the hypomethylated DMRs (odds ratio = 1.6; fisher’s exact test *p* value = 0.0028; Fig. 3D). Next, we explored potential functional characteristics of target genome segments with above DMRs using the data of ENCODE (http://genome.ucsc.edu/ENCODE/) and published potential regulatory motif [21]. The results showed that 173 DMRs (28.1%) had potential promoter, enhancer and/or insulator functions, whereas 128 DMRs (20.4%) had potential transcription factor binding site, and 57 DMRs (9.1%) contained potential regulator motif (Table 1), suggesting that these DMRs likely had functional potential to regulate gene transcription.

**Table 1:** The DMRs overlapped with potential genomic functional sequences.

	Regulatory motif	Transcription factor binding site	Chromatin state (promoter, enhancer, insulator)
DMRs	57 (9.1%)	128 (20.4%)	173 (28.1%)

### DMRs are enriched in genes associated with age-related diseases

Based on the human annotation information, the identified DMRs locate in 251 genes (S2 Dataset). Gene ontology (GO) analysis showed that the hypermethylated genes were mainly involved in several important biological processes, such as developmental processes (*p* = 2.64 × 10^-5^), cell adhesion (*p* = 3.42 × 10^-5^), signal transduction (*p* = 5.94 × 10^-5^) and cell communication (*p* = 1.77 × 10^-4^), whereas the hypomethylated genes were enriched in signal transduction (*p* = 6.27 × 10^-6^), cell communication (*p* = 9.92 × 10^-6^) and also cell adhesion (*p* = 8.27 × 10^-6^) (Table 2). Moreover, pathway analysis revealed that these genes were significantly enriched in several signaling pathways with the hepermethylated genes in Cadherin signaling (*p* = 1.54 × 10^-7^) and Wnt signaling pathways (*p* = 2.19 × 10^-7^); while the hypomethylated genes were enriched in Alzheimer disease-presenilin (*p* = 1.08 × 10^-4^) and Cadherin signaling pathways (*p* = 2.67 × 10^-3^) (Table 3).

**Table 2:** Gene Ontology enrichment analysis for the genes with DMRs in Chinese samples.

GO term	Description	P value
Hypermethylated		
GO:0016337	cell-cell adhesion	9.74E-07
GO:0009790	embryo development	4.66E-06
GO:0007399	nervous system development	5.86E-06
GO:0032502	developmental process	2.64E-05
GO:0007155	cell adhesion	3.42E-05
GO:0007398	ectoderm development	5.47E-05
GO:0007165	signal transduction	5.94E-05
GO:0048731	system development	9.27E-05
GO:0007154	cell communication	1.77E-04
GO:0009987	cellular process	3.85E-04
Hypomethylated		
GO:0009987	cellular process	3.82E-06
GO:0007165	signal transduction	6.27E-06
GO:0007155	cell adhesion	8.27E-06
GO:0007154	cell communication	9.92E-06
GO:0016337	cell-cell adhesion	1.10E-04
GO:0007398	ectoderm development	1.36E-04
GO:0007399	nervous system development	1.54E-04

**Table 3:** Pathway enrichment analysis for the genes with DMRs in Chinese samples.

Pathway	P value
Hypermethylated	
Cadherin signaling pathway	1.54E-07
Wnt signaling pathway	2.19E-07
Hypomethylated	
Alzheimer disease-presenilin pathway	1.08E-04
Cadherin signaling pathway	2.67E-03

**Fig 4:**
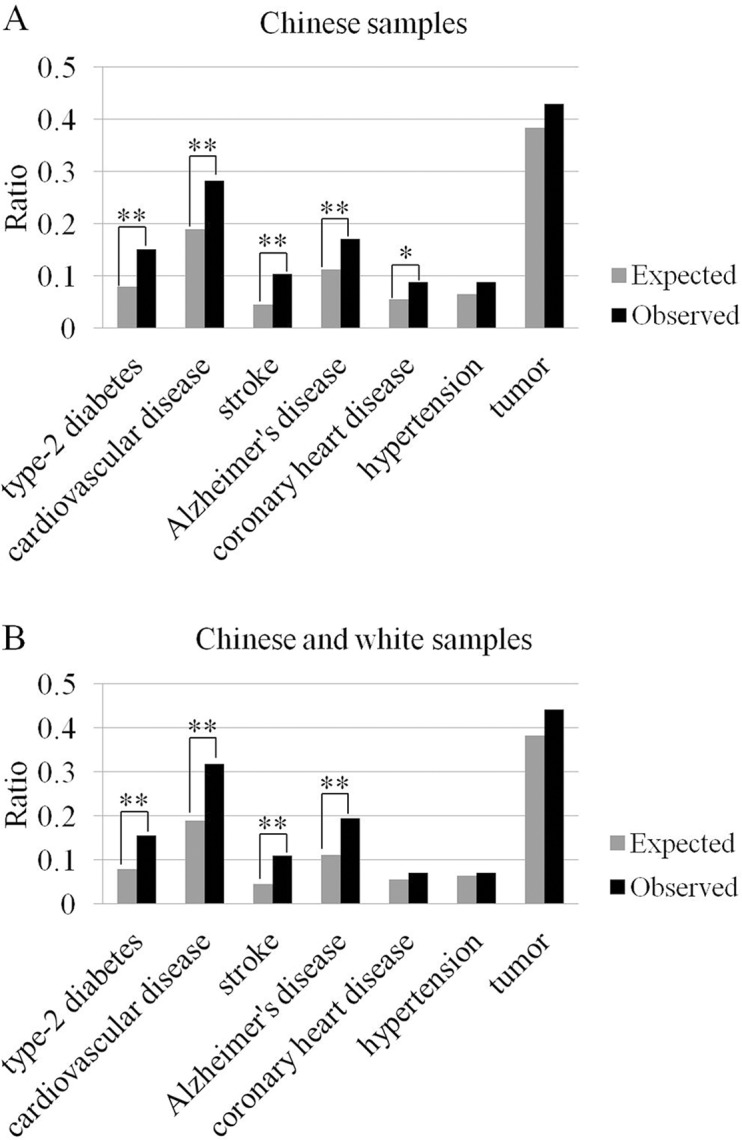
Gene set enrichment analysis (* p < 0.05; ** p < 0.01). (A) The gene set with DMRs in Chinese samples has a significant enrichment in age-related disease genes, including type-2 diabetes, cardiovascular disease, stroke, Alzheimer’s disease and coronary artery disease. (B) The gene set with DMRs in both Chinese and white samples is also enriched in age-related disease genes.

Since these pathways show close relationship with age-related diseases such as Alzheimer’s disease, cardiovascular disease, diabetes mellitus and cancer [22–26], to test whether the genes with DMRs are enriched in age-related diseases, we further conducted an analysis for the associations of differentially methylated genes with diseases. The result revealed that these genes did be significantly enriched in type-2 diabetes (*p* = 4.95 × 10^-5^), stroke (*p* = 2.57 × 10^-5^), cardiovascular disease (*p* = 1.19 × 10^-4^), Alzheimer’s disease (*p* = 1.81 × 10^-3^), and coronary artery disease (*p* = 1.24 × 10^-2^) (Fig. 4A).

To test whether our observation could be replicated, the reported whole genome bisulfite sequencing data from white people [including a male centenarian (Y103) and a male middle-aged subject (Y26) [19]] were included for reanalysis. Consistent with our former observation that most of the DMRs were in intronic and intergenic regions, a total of 14,177 hyper-DMRs and 21,720 hypo-DMRs were identified in promoter (6.5%), exonic (16.9%), intronic (48.8%) and intergenic (45.1%) regions. Similarly, 154 genes with DMRs were observed in both Chinese and white centenarians compared to their middle-aged controls (S3 Dataset), and the enrichment analysis also showed that the 154 genes were enriched on biological process of cell adhesion (*p* = 1.08 × 10^-9^), and pathways of Cadherin (*p* = 1.71 × 10^-8^) and Wnt signaling (*p* = 1.28 × 10^-7^) (S1 and S2 Tables). Intriguingly, the differentially methylated genes were also observed to be overrepresented in type-2 diabetes (*p* = 6.23 × 10^-11^), cardiovascular disease (*p* = 4.49 × 10^-5^), stroke (*p* = 1.96 × 10^-4^) and Alzheimer’s disease (*p* = 8.74 × 10^-4^) (Fig. 4B).

## Discussion

Hitherto, understanding of the genetic mechanism of human longevity remains highly controversial, with one but prevalent hypothesis suggesting the existence of longevity genes whereas another simply attributing it to the lack of diseases-susceptibility mutations. The latter hypothesis, although well explains the low prevalence of age-related diseases in the long-lived people, finds no support from the recent genetic studies [10, 11]. These observations seem to argue for the longevity-gene model, however, taking into consideration the crucial role of epigenetic modification in gene regulation, it remains plausible that suppressing the disease-related genes in the longevity individuals could be achieved by the epigenetic modification, e.g. DNA methylation.

In the present study, by obtaining the genome-wide landscapes of DNA methylation in Chinese centenarians and middle-aged controls and then identifying their differentially methylated regions (DMRs), our results did show that the identified DMRs were significantly enriched in genes associated with age-related diseases, such as type-2 diabetes (*p* = 4.95 × 10^-5^), stroke (*p* = 2.57 × 10^-5^), cardiovascular disease (*p* = 1.19 × 10^-4^) and Alzheimer’s disease (*p* = 1.81 × 10^-3^). Intriguingly, this pattern remained rather stable after the epigenetic genomes from the white centenarian and younger samples [19] were included. Indeed, when looking further into the expression pattern of the genes containing DMRs, we did find some interesting clues. For instance, the Alzheimer’s disease-associated gene *CASP3* shows high expression in the patients [27, 28], which however has a hypermethylated DMR near its transcription start site in centenarians. Similarly, *IL1R2* gene has a lower expression in atherosclerotic disease [29, 30] but contains a hypo-DMR near its transcription start site in our centenarians. These results likely reflect a functional role of the observed DMRs in regulating the expression of some disease-associated genes, with some genes (e.g. *CASP3*) being down-regulated whereas the others (i.e. *IL1R2*) up-regulated. Taken together, these observations seem to be in well agreement with the ability of centenarians in suppressing or escaping the age-related diseases [7, 12, 31].

Although further efforts are needed to shed light on the genuine function of the observed DMRs in our longevity samples, it is difficult to simply attribute their significant enrichment (*p* < 0.05) on the genes associated with age-related diseases to be a random process because of three reasons. First, this enrichment pattern keeps rather stable even the white samples, which are known to have quite different genetic backgrounds from the Chinese [32–34], were included for analysis. Second, much lower prevalence of the age-related diseases in the centenarians is reported by more and more epidemiological surveys [6, 7]. Third, some clues, albeit rather meager at the current stage, between the distilled DMRs and the expression of the susceptibility genes did have been observed, which shall become more pronounced if the methylome and transcriptome data from the same samples are obtained. Taken together, it is then most likely that DNA methylation may contribute to healthy aging in human populations by regulating the genes susceptible to the age-related diseases.

In short, our genome-wide scan does reveal a large number of DMRs existing between the centenarians and younger control subjects, which likely play an important but previously unrecognized role in regulating the genes, especially those that show susceptibility to the age-related diseases. These observations seem to be in accordance with the ability of centenarians in escaping or delaying the age-related diseases. Therefore, our study suggests that suppressing the disease-related genes via the epigenetic modification is likely an important contributor in human longevity.

## Materials and Methods

### Methylated DNA immunoprecipitation and Illumina Genome Analyzer sequencing

We collected peripheral blood from 4 centenarians and 4 middle-aged controls from four different provinces in China (S3 Table). Investigation has been conducted in accordance with the ethical standards and according to the Declaration of Helsinki and according to national and international guidelines and has been approved by the review board at Kunming Institute of Zoology, Chinese Academy of Sciences. Written informed consent was obtained from each of the participants prior to the study. All of them were local native residents. Approximately 5 μg of DNA from each sample was used for MeDIP-seq library construction as described by Li *et al*. [18] The genomic DNA was sonicated into random fragments ranging from 100–500 bp. Finally, 49 bp paired-end reads were produced for the methylation profile analysis by next generation sequencing.

### Mapping reads and identification of DMRs

We mapped the raw reads onto human genome hg18 build, which was downloaded from the University of California Santa Cruz (UCSC) Bioinformatics Site (http://genome.ucsc.edu/), using the alignment software SOAPaligner v2.21 (http://soap.genomics.org.cn/) with no more than 2 bp mismatched [35]. Here we considered the length of sequenced MeDIP-enriched DNA fragments as 400 bp and thus extended the uniquely mapping short reads to 400 bp to represent the real methylated DNA fragments (S4 Table).

Then, we divided the entire genome into 200 bp non-overlap segments and counted the number of reads mapped within each segment. The segments covered at least by 1 read in each sample and more than 10 reads for the mean depth of 8 samples were used for further study. The methylation profile data were analyzed to find the genomic regions with different methylation status between the centenarian group and younger group using the edgeR package based on the reads number of each segment [36].

### Analysis of published whole genome bisulfite sequencing data

Two white individuals’ WGBS data [one 103-year-old white man (Y103) and one 26-year-old white man (Y26)] [19] were downloaded from NCBI (http://www.ncbi.nlm.nih.gov/) with GSM774849 and GSM848927. The methylation level of each CpG was calculated using the Bismark [37]. The CpGs covered less than 5 reads were first removed and then the differentially methylated CpGs were called based on the methylated and unmethylated numbers of reads in the two white individuals (fisher’s exact test, *p* < 0.05; the methylation level of the CpGs between Y103 and Y26 with a minimum difference of > 20%). Then adjacent differentially methylated CpGs (the distance between two CpGs less than 1000 bp) with a same directional change were merged as a big segment and only those segments with no less than 5 CpGs were chosen as final DMRs.

### Genome annotation information

The human gene annotation information hg18 build was downloaded from Ensembl database (http://www.ensembl.org/). The promoter regions were defined as 2k bp of upstream region of the transcription start sites in Ensembl database. The human chromatin state and genomic transcription factor binding sites data were downloaded from ENCODE (http://genome.ucsc.edu/ENCODE/).

### Gene set enrichment analysis

Using the Protein Analysis Through Evolutionary Relationships (PANTHER) Classification System 8.1 [38], gene ontology biological process and pathway were analyzed. Moreover, gene lists related with age-related diseases (e.g. Alzheimer’s disease, type-2 diabetes, cardiovascular disease) were got from GeneCards version 3.11 [39], and a hypergeometric test was conducted to find the enriched disease terms based on the observed and expected gene numbers.

### Statistical analysis

The statistic methods, like hypergeomeric test and fisher’s exact test, were calculated using the *phyper* and *fish*.*test* function provided within the R framework (http://www.R-project.org/).

## Supporting Information

S1 Dataset The identified DMRs in Chinese samples. (XLS)

S2 Dataset The genes with the DMRs in Chinese samples. (XLS)

S3 Dataset The genes with DMRs in both Chinese and white samples. (XLS)

S1 Fig Saturation analysis. The result showed that our data can generate a reproducible methylation profile for each sample.
(TIF)

S2 Fig Coverage analysis. The result showed that our data can cover more than 80% CpGs in human genome.
(TIF)

S1 Table Gene Ontology enrichment analysis for genes with DMRs in both Chinese and white samples. The genes were enriched in cell adhesion and development-related GO terms.
(DOC)

S2 Table Pathway enrichment analysis for genes with DMRs in both Chinese and white samples. The genes were enriched in Cadherin and Wnt signaling pathway.
(DOC)

S3 Table Sample information. (DOC)

S4 Table Reads mapping. More than 60 million uniquely mapped paired-end reads were produced for each sample.
(DOC)
