# Supplementary figures and images for: A Genome-Wide Scan Reveals Important Roles of DNA Methylation in Human Longevity by Regulating Age-Related Disease Genes
Source: PLoS One. 2015 Mar 20;10(3):e0120388. doi: 10.1371/journal.pone.0120388 (PMC4368809; doi:10.1371/journal.pone.0120388)

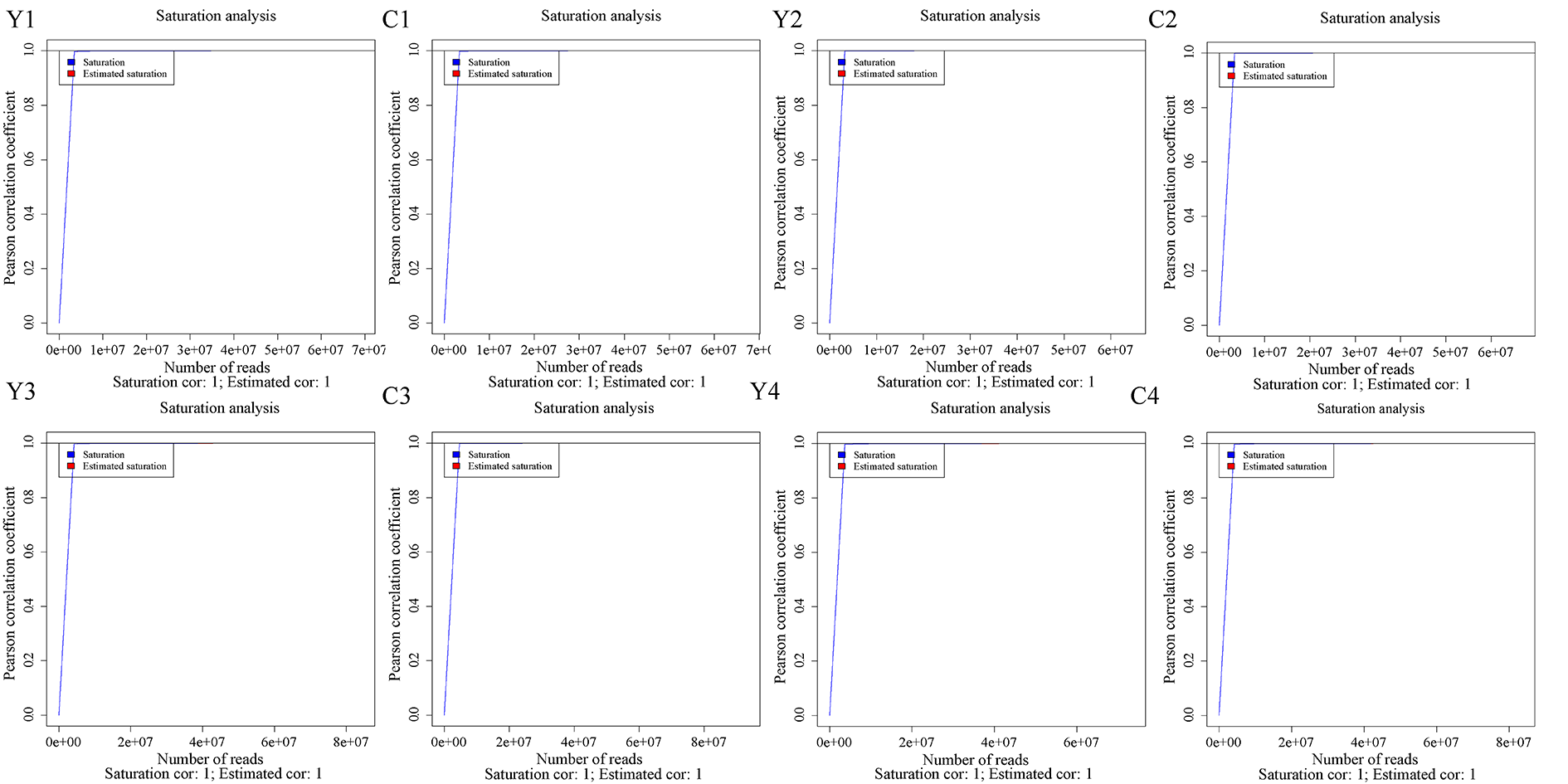

Supplement: S1 Fig — The result showed that our data can generate a reproducible methylation profile for each sample. (TIF) [file pone.0120388.s004.tif]

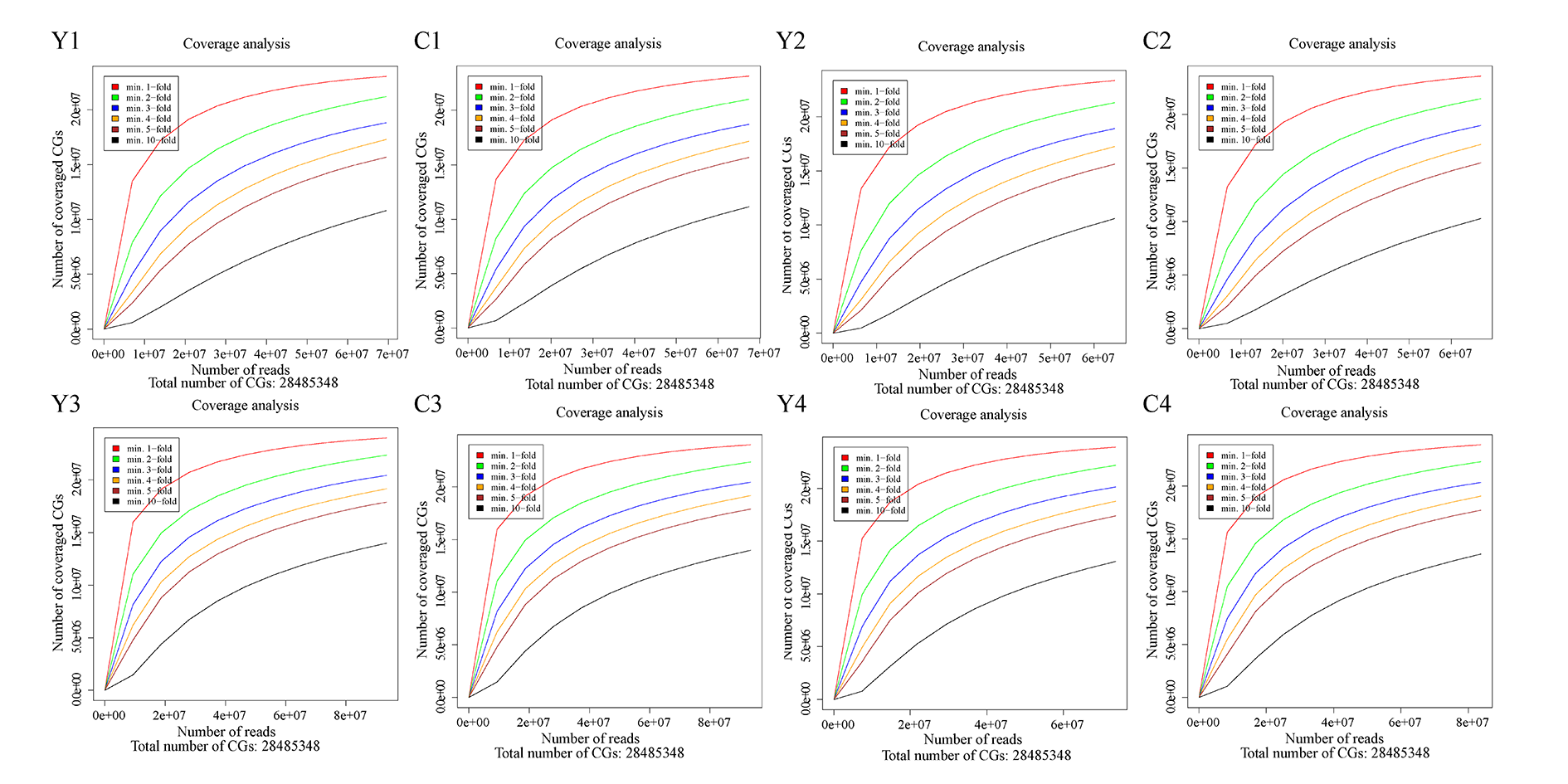

Supplement: S2 Fig — The result showed that our data can cover more than 80% CpGs in human genome. (TIF) [file pone.0120388.s005.tif]
