# Supplementary material for: A Genome-Wide Scan Reveals Important Roles of DNA Methylation in Human Longevity by Regulating Age-Related Disease Genes
Source: PLoS One. 2015 Mar 20;10(3):e0120388. doi: 10.1371/journal.pone.0120388 (PMC4368809; doi:10.1371/journal.pone.0120388)
Supplement: S1 Table — The genes were enriched in cell adhesion and development-related GO terms. (DOC) [file pone.0120388.s006.doc]

**S1 Table. Gene Ontology enrichment analysis for genes with DMRs in both Chinese and white samples. The genes were enriched in cell adhesion and development-related GO terms.**

| GO term | Description | P value |
| --- | --- | --- |
| GO:0007155 | cell adhesion | 7.69E-10 |
| GO:0016337 | cell-cell adhesion | 1.05E-09 |
| GO:0007399 | nervous system development | 4.08E-08 |
| GO:0009987 | cellular process | 5.55E-08 |
| GO:0007398 | ectoderm development | 5.17E-07 |
| GO:0007154 | cell communication | 2.01E-06 |
| GO:0048731 | system development | 2.48E-06 |
| GO:0032502 | developmental process | 3.87E-06 |
