# Supplementary material for: A Genome-Wide Scan Reveals Important Roles of DNA Methylation in Human Longevity by Regulating Age-Related Disease Genes
Source: PLoS One. 2015 Mar 20;10(3):e0120388. doi: 10.1371/journal.pone.0120388 (PMC4368809; doi:10.1371/journal.pone.0120388)
Supplement: S2 Table — The genes were enriched in Cadherin and Wnt signaling pathway. (DOC) [file pone.0120388.s007.doc]

**S2 Table. Pathway enrichment analysis for genes with DMRs in both Chinese and white samples. The genes were enriched in Cadherin and Wnt signaling pathway.**

| Pathway | P value |
| --- | --- |
| Cadherin signaling pathway | 6.97E-09 |
| Wnt signaling pathway | 1.19E-08 |
