# Supplementary material for: A Genome-Wide Scan Reveals Important Roles of DNA Methylation in Human Longevity by Regulating Age-Related Disease Genes
Source: PLoS One. 2015 Mar 20;10(3):e0120388. doi: 10.1371/journal.pone.0120388 (PMC4368809; doi:10.1371/journal.pone.0120388)
Supplement: S3 Table — (DOC) [file pone.0120388.s008.doc]

**S3 Table. Sample information.**

| Sample ID | Age (years) | Ethnic group | Sex | Region (province) |
| --- | --- | --- | --- | --- |
| Y1 | 46 | Zhuang | Female | Guangxi |
| C1 | 106 |  |  |  |
| Y2 | 40 | Dai |  | Yunnan |
| C2 | 101 |  |  |  |
| Y3 | 43 | Li |  | Hainan |
| C3 | 100 |  |  |  |
| Y4 | 43 | Han |  | Sichuan |
| C4 | 104 |  |  |  |

Notes: Y and C represent younger control and centenarian samples, respectively
