# Supplementary material for: A Genome-Wide Scan Reveals Important Roles of DNA Methylation in Human Longevity by Regulating Age-Related Disease Genes
Source: PLoS One. 2015 Mar 20;10(3):e0120388. doi: 10.1371/journal.pone.0120388 (PMC4368809; doi:10.1371/journal.pone.0120388)
Supplement: S4 Table — More than 60 million uniquely mapped paired-end reads were produced for each sample. (DOC) [file pone.0120388.s009.doc]

**S4 Table. Reads mapping. More than 60 million uniquely mapped paired-end reads were produced for each sample.**

| Sample ID | Read length (bp) | Raw reads number | Mapped reads number | Mapping rate (%) | Uniquely mapped reads number | Uniquely mapping rate (%) |
| --- | --- | --- | --- | --- | --- | --- |
| Y1 | 49 PE | 97,959,184 | 85,450,938 | 87.23 | 69,515,451 | 70.96 |
| C1 |  | 97,943,824 | 85,142,917 | 86.93 | 67,465,288 | 68.88 |
| Y2 |  | 97,959,184 | 84,259,507 | 86.01 | 64,787,210 | 66.14 |
| C2 |  | 97,959,184 | 84,940,252 | 86.71 | 67,010,979 | 68.41 |
| Y3 |  | 118,721,966 | 103,104,911 | 86.85 | 84,502,981 | 71.18 |
| C3 |  | 133,795,892 | 117,944,018 | 88.16 | 93,476,766 | 69.87 |
| Y4 |  | 102,228,158 | 91,147,868 | 89.16 | 73,699,953 | 72.09 |
| C4 |  | 114,709,826 | 102,149,508 | 89.05 | 83,738,350 | 73.00 |

Notes: Y and C represent younger control and centenarian samples, respectively; PE, paired-end
